# The effect of vitamin D status on different neuromuscular blocker agents reverse time

**DOI:** 10.3906/sag-1901-115

**Published:** 2020-06-23

**Authors:** İlknur Suidiye YORULMAZ, Yavuz DEMİRARAN, Onur ÖZLÜ, Burhan DOST

**Affiliations:** 1 Department of Anesthesiology and Reanimation, Faculty of Medicine, Düzce University, Düzce Turkey; 2 Department of Anesthesiology and Reanimation, Medipol University Mega Hospital Complex, İstanbul Turkey; 3 Department of Anesthesiology and Reanimation, TOBB Economy and Technology University, Faculty of Medicine, Ankara Turkey; 4 Department of Anesthesiology and Reanimation, Ondokuz Mayıs University, Samsun Turkey

**Keywords:** Anesthesia, vitamin D, muscle relaxation, sugammadex, neostigmine, neuromuscular blocker

## Abstract

**Background/aim:**

This study is aimed to investigate the effects of vitamin D levels on sugammadex and neostigmine reversal times.

**Material and methods:**

Eighty patients between the ages of 18 and 65 years, with ASA I-III status who were undergoing surgery under general anesthesia were included in the study. A double blind fashion was used to randomly divide all the patients into two groups. At the end of the operation, sugammadex 2 mg/kg was administered to one group (Group sugammadex) and atropine and neostigmine was administered to the other group (Group neostigmine) intravenously. In the data analysis stage, the group was divided into two subgroups according to sugammadex and group neostigmine in itself, with vitamin D levels above and below 30 ng/mL. Statistical analysis was performed on these 4 groups (Group neostigmine and vitamin D < 30 ng/mL), (Group neostigmine and vitamin D ≥ 30 ng/mL), ( Group sugammadex and vitamin D < 30 ng/mL), (Group sugammadex and vitamin D ≥ 30 ng/mL).When two responses to train of four (TOF) stimulation were taken, the following times were recorded until extubation phase. The time until TOF value 50%, 70%, 90%, and extubation were recorded.

**Results:**

There were statistically significant differences between Group sugammadex and vitamin D < 30 ng/mL and Group sugammadex and vitamin D ≥ 30 ng/mL (P = 0.007) for extubation times and 50% TOF reach times (P = 0.015). However, there was no difference observed between Group neostigmine and vitamin D < 30 ng/mL and Group neostigmine and vitamin D ≥ 30 ng/mL (P = 0.999).

**Conclusion:**

Vitamin D deficiency is important for anesthesiologists in terms of muscle strength and extubation time. Vitamin D deficiency seems to affect sugammadex reverse times but seems not to affect neostigmine reverse times. This conclusion needs further studies.

## 1. Introduction

Vitamin D is essential for intestinal calcium absorption and for maintaining calcium homeostasis. Calcium draws on the release of acetylcholine from the motor nerve terminal and increases the excitation–contraction coupling in the muscle. In addition, calcium plays an important role for contraction in skeletal muscles [1]. Vitamin D refers to vitamin D2 (ergocalciferol) or vitamin D3 (cholecalciferol). Vitamin D3 is made in the skin or obtained in the diet (D2 or D3), it enters the circulation and is bound to vitamin D binding protein. This complex is transported to the liver and after hydroxylation becomes 25-hydroxyvitamin D (25[OH] D). Then in kidney, it is hydroxylated by 1- alfa hydroxylase and takes the form of 1,25-dihydroxyvitamin D (1,25[OH] D). Vitamin D binding protein and 1,25-dihydroxyvitamin D binds with each other and vitamin D enters the target cells. In the target cell cytoplasm vitamin D binds with vitamin D receptor and enters the nucleus. In the nucleus vitamin D and retinoic acid heterodimers increase the transcription of vitamin D-dependent genes important for bone metabolism and calcium absorption [2]. Anticholine esterases show their effect by inhibiting acetylcholinesterase and butyrylcholinesterase by prolonging the existence of acetylcholine at the motor end-plate [3]. Neostigmine is the most preferred anticholine esterase drug [4]. Sugammadex forms complexes with steroidal neuro-muscular blocking agents (NMBAs) (rocuronium > vecuronium ≫ pancuronium) and helps in the rapid removal of free rocuronium [5]. Neuromuscular transmission is clinically monitored using the train-of-four (TOF) ratio, which is the quotient between twitch tension produced by the fourth (T4) and by the first (T1) stimulus within a TOF stimulation at 2 Hz.

We have started to arouse curiosity among anesthesiologists who work with muscle relaxant agents as to whether the changes in vitamin D levels are among the factors. In the literature, we did not encouter any study for neuromuscular agent reverse durations and vitamin D levels. Moreover, there is no study in the literature reporting the effect of vitamin D levels on the onset and return of neuromuscular blocker agents by the use of TOF ratio. This study is aimed to investigate the effect of vitamin D levels on sugammadex and neostigmine reversal times.

## 2. Materials and methods

This prospective, randomized, observational study was carried out in the same center after the approval of the School of Medicine of Düzce University by the ethics committee of noninvasive research (Düzce University, School of Medicine, Ethics Committee of Noninvasive clinical research approval for application Date: 11.10.2013, Decision Number: 2013/439) and was done by the Anesthesiology Department in the same center. This study was registered to the ClinicalTrials.gov (Identifier: NCT03734250). 

The inclusion criteria were to be between 18 and 65 years old, American Society of Anesthesiologists (ASA) I-III status, and operated for elective surgery under general anesthesia. The patients with hypothermia, neuromuscular diseases, hypothyroidism, hyperthyroidism, diabetes mellitus, and electrolyte imbalance were excluded. Written informed consent was taken from all of the patients.

### 2.1. Anesthesia management

In the operating room, all the patients were monitored using a standard monitoring procedure (heart rate, noninvasive arterial pressure, pulse-oximetry, end tidal carbon dioxide) and neuromuscular block monitoring were performed. After patients had an intravenous line performed in the back of the hand with a 20 gauge, first 10 mL blood samples were taken and then intravenous fluids were administered. The 25-hydroxyvitamin D (25(OH) Vitamin D ELISA kit, abcam, UK), calcium, magnesium, phosphorus and parathormone levels were measured in the blood samples. A double blind fashion was used to randomly divide all the patients into two groups: (Group sugammadex; n = 42), (Group neostigmine; n = 42). 

Neuromuscular monitoring was performed to all patients by connecting to a kinemyograph, which placed the neuromuscular transmission (NMT) module (E-NMT module, GE Healthcare Finland Oy, Helsinki, Finland) on the patients’ adductor pollicis muscle before the induction of anesthesia. Patients’ body temperatures were maintained within 35–37 °C. All the patients received midazolam 2 mg intravenously for premedication. All the patients were received propofol 2 mg/kg and fentanyl 1 µg/kg. Subsequently, propofol was given at the beginning of the operation and TOF values were recorded as basal values (TOFbasal) and during the operation. TOF stimulation started at 1 Hz intervals at a frequency of 2 Hz in 1-min intervals. The initial values determined were recorded as the baseline TOF values and rocuronium 0.5 mg/kg (Esmeron amp, Organon, Holland) was administered intravenously, which was calculated for ideal body weight. The time to complete the disappearance of response to TOF stimulation was recorded as T0 and endotracheal intubation was performed.

Anesthetic maintenance was proven by 50% oxygen, 50% air, and sevoflurane 2% via endotracheal tube. Remifentanil hydrochloride (Ultiva flk, Aspen Pharma Trading Limited, Ireland) infusion was administered 0.05–1 µg/kg / h intravenously. The muscle relaxant was administered in repeated doses as 10 mg of rocuronium when a 2-point response to TOF was received (Posttetanic count (PTC) < 10). 

The total muscle relaxant amounts were recorded. Patients were divided into two groups randomly and sugammadex (Group sugammadex; n = 42) and neostigmine (Group Neostigmine; n=42 ) were administered intravenously. At the end of the surgery: when two responses were achieved from TOF stimulation, sugammadex sodium 2 mg/kg (Bridion flk, Merck Sharp & Dohme, U.K) or neostigmine 0.05 mg/kg (Neostigmin amp, Adeka, Turkey) and Atropine 0.015 mg/kg (Atropine sulphate, Galen, Turkey) per kg body weight were performed to the patient. Data were recorded as time to reach 50%, 70%, 90% TOF values and extubation. In the data analysis stage, each group was divided into two subgroups according to sugammadex and group neostigmine in itself, with vitamin D levels above and below 30 ng/mL. Statistical analysis was performed on these four groups. (Group neostigmine and vitamin D < 30 ng/mL), (Group neostigmine and vitamin D ≥ 30 ng/mL), (Group sugammadex and vitamin D < 30 ng/mL), (Group sugammadex and vitamin D ≥ 30 ng/mL).

TOF0: The time between rocuronium administration and TOF disappearance.

TOF0.5: The time until the TOF value reached 50%.

TOF0.7: The time until the TOF value reached 70%.

TOF0.9: The time until the TOF value reached 90%.

TOFext: Time to extubation.

TOFbasal: TOF values which measured after propofol administration.

Extubation criteria: Patients who were able to open their eyes with spontaneous breathing, had a tidal volume 10 mL/kg, according to ideal body weight during spontaneous breathing, pulse oximetry values above 95%, ability to head lift for 5 s, and TOF values above 90%.

### 2.2. Labarotory protocol

Blood samples were centrifuged at 2000 rpm for 10 min in a refrigerated centrifuge to separate serum samples. All samples were stored at –70 °C until they were analyzed. Serum total 25-hydroxyvitamin D assay was performed using ELISA kits.

### 2.3. Sample size and power analysis

 The proposed sample size was calculated via power analyses using GPower 3.1 software. Sample size of 20 was determined for each group with a priori power analyses, according to the effects size, power analysis and sample size test for medium effect size=0.45 alpha=0.05 and test power of 80%. Although a minimum of 20 participants per condition is recommended, 21 participants are assigned in each group to account for potential erroneous or other unexpected problems [6].

### 2.4. Statistical analysis

Statistical analyses were performed with the SPSS v.22 package program and the significance level was accepted as P < 0.05. Normal distribution of the TOF variables was checked with the Kolmogorov–Smirnov test (statistic; P for basal, 50%, 70%, 90% extubation and disappearance of TOF values and 0.954; 0.189, 0.974; 0.620, 0.969; 0.476, 0.899; 0.069, 0.973; 0.596; and 0.959; 0.266, respectively). As normal distribution of variables was ensured, we continued data analysis with parametric tests. The independent samples t-test for two variables was used for comparison of continuous variables. For categorical variables, the Pearson Chi-square test was used. Repeated measures ANOVA was used to examine the interaction between the drug groups and TOF measurement time. Coeffects of drug groups and vitamin D subgroups were examined by two-way analysis of variance (ANOVA). Repeated measures ANOVA, one of the three-factor differentials, was used to examine the TOF measurement time interaction of drug effects and vitamin D subgroups’ concerted effects.

## 3. Results

Although there were no statistically significant differences in age, body mass index (BMI), and height parameters between the sugammadex group and neostigmine group, it was observed that the operation period of the neostigmine group was longer (P = 0.031; Table 1).

**Table 1:** There is no difference of the demographic data between the two groups. Operation time is longer at neostigmine administration group than sugammadex administration group.

	Neostigmine(n=42)	Sugammadex(n=42)	P
Male	21 (50%)	27 (64.3%)	0.186
Female	21 (50%)	15 (35.7%)	
Age (year)	36.6 ± 13.8	38.5 ± 12.2	0.520
Body mass index (kg/m2)	25.8 ± 6.1	27.9 ± 5.6	0.094
Operation time spent (minute)	93.1 ± 43.9	74.7 ± 31.5	0.031

### 3.1. Comparison by group and vitamin D level

Based on the variation of vitamin D (25), we formed two subgroups < 30 ng/mL and ≥ 30 ng/mL. Statistical analysis was performed on these four groups: (Group neostigmine and vitamin D < 30 ng/mL, n=31), (Group neostigmine and vitamin D ≥ 30 ng/mL, n=11), (Group sugammadex and vitamin D < 30 ng/mL, n=37), (Group sugammadex and vitamin D ≥ 30 ng/mL, n=5).

The most important problem here was that the number of patients in the vitamin D ≥ 30 ng/mL group and the number of patients in the quadruple group were unbalanced (As we mentioned above).

For both groups, the main effect (The difference between the groups in terms of mean age does not change according to whether vitamin D is low or high.) (P = 0.671) and the low/high vitamin D group main effect (P = 0.611) were not significant. All the groups were evaluated as homogeneous and there was no difference in age.

The calcium, magnesium, phosphor levels, and the interaction between the groups and vitamin D status were given in Table 2. 

**Table 2:** Calcium, magnesium, phosphor, and parathormone levels of the groups.* Phosphor levels are higher in group sugammadex.

	GroupNeostigmine (n=42)	GroupSugammadex (n=42)	P
Calcium (mg/dL)	8.7 ± 0.9	9.1 ± 1.1	0.073
Magnesium (mg/dL)	1.9 ± 0.3	2.0 ± 0.2	0.187
Phosphor (mg/dL)	2.8 ± 0.6	3.1 ± 0.7	0.016*
Parathormone (pg/mL)	72.6 ± 35.0	63.6 ± 28.7	0.204

### 3.2. Comparison of TOF values

(Group neostigmine and vitamin D < 30 ng/mL, n=31), (Group neostigmine and vitamin D ≥ 30 ng/mL, n=11), (Group sugammadex and vitamin D < 30 ng/mL, n=37), (Group sugammadex and vitamin D ≥ 30 ng/mL, n=5).

#### 3.2.1. Basic measurement 

There was no difference between Group neostigmine and vitamin D < 30 ng/mL, n=31 and Group neostigmine and vitamin D ≥ 30 ng/mL, (P = 0.999).

 There was no difference between Group sugammadex and vitamin D < 30 ng/mL, n=31 and Group sugammadex and vitamin D ≥ 30 ng/mL, (P = 0.999).

#### 3.2.2. 50% measurement

There was no difference between (Group neostigmine and vitamin D < 30 ng/mL, n=31) and (Group neostigmine and vitamin D ≥ 30 ng/mL, n=11), (P = 0.999). 

There was a significant statistical difference between (Group sugammadex and vitamin D < 30 ng/mL, n=37) and (Group sugammadex and vitamin D ≥ 30 ng/mL, n=5),

 (P = 0.015). 

There was no difference between (Group neostigmine and vitamin D < 30 ng/mL, n=31) and Group sugammadex and vitamin D < 30 ng/mL, n=37) (P = 0.999). 

There was no difference between (Group neostigmine and vitamin D ≥ 30 ng/mL, n=11) and (Group sugammadex and vitamin D ≥ 30 ng/mL, n=5) (P = 0.987).

#### 3.2.3. 70% measurement 

There was no difference between (Group neostigmine and vitamin D < 30 ng/mL, n=31) and (Group neostigmine and vitamin D ≥ 30 ng/mL, n=11) (P = 0.999). 

There was no difference between (Group sugammadex and vitamin D < 30 ng/mL, n=37) and (Group sugammadex and vitamin D ≥ 30 ng/mL, n=5), (P = 0.990). 

There was a significant statistical difference between (Group neostigmine and vitamin D < 30 ng/mL, n=31) and (Group sugammadex and vitamin D < 30 ng/mL, n=37) (P < 0.001). 

There was no difference between (Group neostigmine and vitamin D ≥ 30 ng/mL, n=11) and (Group sugammadex and vitamin D ≥ 30 ng/mL, n=5) (P = 0.999).

#### 3.2.4. 90% measurement

There was no difference between (Group neostigmine and vitamin D < 30 ng/mL, n=31) and (Group neostigmine and vitamin D ≥ 30 ng/mL, n=11) (P = 0.999). 

There was no difference between (Group sugammadex and vitamin D < 30 ng/mL, n=37) and (Group sugammadex and vitamin D ≥ 30 ng/mL, n=5) (P = 0.990).

 There was a significant difference between (Group neostigmine and vitamin D < 30 ng/mL, n=31) and (Group sugammadex and vitamin D < 30 ng/mL, n=37) (P < 0.001). 

There was no difference between (Group neostigmine and vitamin D ≥ 30 ng/mL, n=11) and (Group sugammadex and vitamin D ≥ 30 ng/mL, n=5) (P = 0.992).

#### 3.2.5. Extubation measurements

There was no difference between (Group neostigmine and vitamin D < 30 ng/mL, n=31) and (Group neostigmine and vitamin D ≥ 30 ng/mL, n=11) (P = 0.999). 

There was a significant difference between (Group sugammadex and vitamin D < 30 ng/mL, n=37) and (Group sugammadex and vitamin D ≥ 30 ng/mL, n=5) (P = .0.007) There was a significant difference between (Group neostigmine and vitamin D < 30 ng/mL, n=31) and (Group sugammadex and vitamin D < 30 ng/mL, n=37) (P = 0.002). 

There was no difference between (Group neostigmine and vitamin D ≥ 30 ng/mL, n=11) and (Group sugammadex and vitamin D ≥ 30 ng/mL, n=5) (P = 0.982).

#### 3.2.6. T0 measurements

There was no difference between (Group neostigmine and vitamin D < 30 ng/mL, n=31) and (Group neostigmine and vitamin D ≥ 30 ng/mL, n=11) (P = 0.999). 

There was no difference between (Group sugammadex and vitamin D < 30 ng/mL, n=37) and (Group sugammadex and vitamin D ≥ 30 ng/mL, n=5) (P = 0.999). 

There was no difference between (Group neostigmine and vitamin D < 30 ng/mL, n=31) and Group sugammadex and vitamin D < 30 ng/mL, n=37) (P = 0.999). 

There was no difference between (Group neostigmine and vitamin D ≥ 30 ng/mL, n=11) and (Group sugammadex and vitamin D ≥ 30 ng/mL, n=5) (P = 0.999).

## 4. Discussion

It is also a major problem to achieve postoperative muscle weakness and to maintain the respiratory functions at least at the level of the preoperative status. Optimization and prevention of the factors that affect the muscle contraction is crucial for the anesthesiologist at the preoperative, perioperative, and postoperative periods. Routine reversal of neuromuscular blockade is common in many countries after surgery under general anesthesia because of the prevention of postoperative residual neuromuscular recurarization [7]. 

We observed that sugammadex reverse times were (especially TOF %50 reaching times and extubation times) prolonged, but there was no change in the neostigmine group with vitamin D below 30 ng/mL. This result constitutes our primary outcome. This important finding could have a role in the ‘cannot intubate, cannot ventilate’ situation, a scenario where anticholinesterases have no effect. 

Vitamin D is transformed from 7-dehydrocholesterol into provitamin D3 under ultraviolet rays on the skin. Provitamin D3 turns into vitamin D3. Serum vitamin D is bound to the bound protein and transported to the liver. 25 (OH) D3 is formed by hydroxylation in the liver and it becomes 1,25 dihydroxy vitamin D in the kidneys. This form is the biologically active form of vitamin D [8]. 

The majority of circulating vitamin D is in the form of 25 (OH) D3. Serum 25 (OH) D3 levels were 1000 higher than 1.25 vitamin D levels; therefore, it is recognized as the most appropriate indicator in determining the level of vitamin D. The active form of 1,25 dihydroxy vitamin D has a half-life of 4-6 h [8]. The evidence indicated that 1,25 dihydroxy vitamin D activates severe secondary pathways in the muscle cell by binding to the membrane receptor. It increases calcium uptake through both calcium-activated calcium channels and calcium-dependent calcium channels within minutes [9].

Vitamin D increases the serum calcium level by the nuclear vitamin D receptor (VDR) [10]. VDRs are found in the muscle tissues. Vitamin D effects and improves by direct effect on muscle tissues itself [11,12]. Vitamin D deficiency affects the faster and stronger type 2 muscle fibers. Hypovitaminosis D impairs neuromuscular coordination [11,12]. Most circulating 25 (OH) D3 and 1,25 dihydroxy vitamin D are transported in the blood bound with vitamin D binding protein (80–90%) and albumin (10–20%), only a small fraction is free or unbound [13]. 

Adequacy of vitamin D levels were defined as a result of various observational studies and analyses with 25 (OH) D3 levels in the serum being above 30 ng/mL (75 nmol/L). Vitamin D deficiency was reported as 21–29 ng/mL (52-72 nmol/l) in 25 (OH) D3 levels in the serum [14]. 

Neostigmine 0.004–0.007 µg/kg has an onset action within 1 min its peak effect does not occur for 9 min [15]. The advised sugammadex dose for reversal of a moderate NMB is 2 mg/kg and sugammadex 4 mg/kg for reversal of a deep NMB. With these doses, a TOF-ratio ≥0.9 is achieved in about 2 min (reversal of moderate NMB) and 1.6–3.3 min (deep NMB) [16]. In contrast, it takes on average 12.8 and 48.8 min to reach a TOF-ratio ≥0.9 when neostigmine 0.05–0.07 mg/kg is used for the reversal of a moderate and deep NMB, respectively [16]. 

Suy and colleagues examined the efficacy of sugammadex 0.5 and 4 mg/kg to reverse modarate neuromuscular block induced by rocuronium group. They found that reappearance of T2, 31.8 min after placebo compared with 3.7 and 1.1 min after sugammadex administration 0.5 and 4 mg/kg respectively [17].

Flocton et al. studied 84 patients who received either rocuronium 0.6 mg/kg and sugammadex 2 mg/kg or cisatracurium 0.15 mg/kg and neostigmine 50 µg/kg to compare sugammadex for the reversal of rocuronium-induced block with that of neostigmine-glycopyrrolate for cisatracurium-induced block. They found that the time recovery of the TOF ratio to 0.9 was faster with sugammadex (2.0 min) than with neostigmine (8.8 min) (P < 0.0001) [18].

In our previous study to compare the time of antagonism and effect of sugammadex (2 mg/kg) which is used for antagonism of rocuronium 0.6 mg/kg on diabetic (n=21) and nondiabetic patients (n=20), we found that extubation time (time from sugammadex administration to TOF ratio reaching 0.9) was 434.6 ± 857.1 [205 (67–4120)] s in diabetics and 250.4 ± 108.4 [247 (106–462)] seconds in nondiabetics (P = 0.948) [19]. 

In our study we found that TOF-ratio 0.9 reach time (437 ± 282 s) for (Group neostigmine and vitamin D < 30 ng/mL, n=31 ), (476 ± 258 s) for (Group neostigmine and vitamin D ≥ 30 ng/mL, n=11). We also found that TOF-ratio 0.9 reach time (151 ± 117 s) for (Group sugammadex and vitamin D < 30 ng/mL, n=37 ), (313 ± 413 s) for (Group sugammadex and vitamin D ≥ 30 ng/mL, n=5) in our study. There is no difference found statistically for TOF-ratio 0.9 reach time between both two groups. 

**Figure 1:**
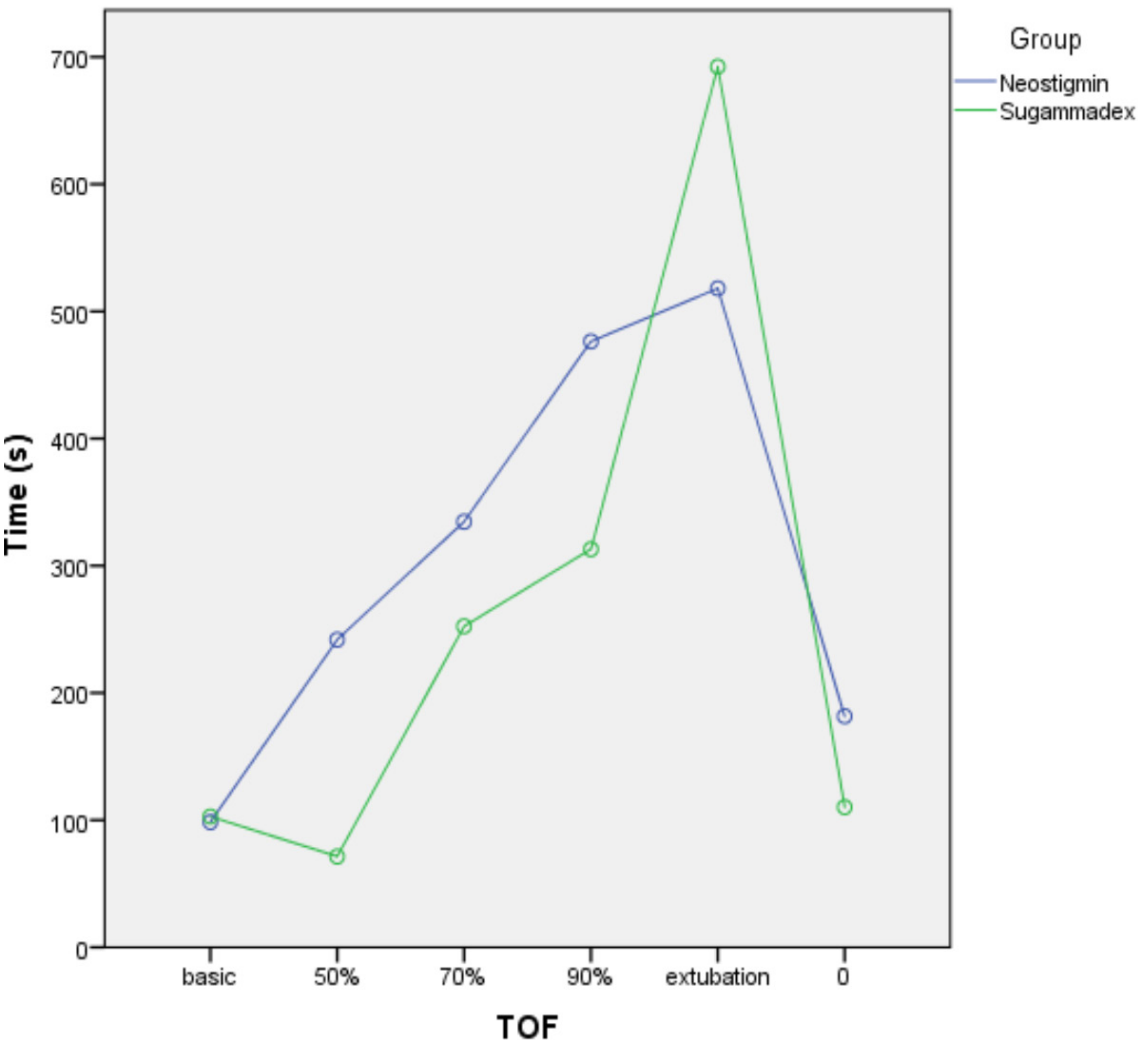
The graphics of the neostigmine (Group neostigmine and vitamin D ≥ 30 ng/mL, n=11) and sugammadex (Group sugammadex and vitamin D ≥ 30 ng/mL, n=5) when vitamin D status is equal or above 30 ng/mL.

**Figure 2:**
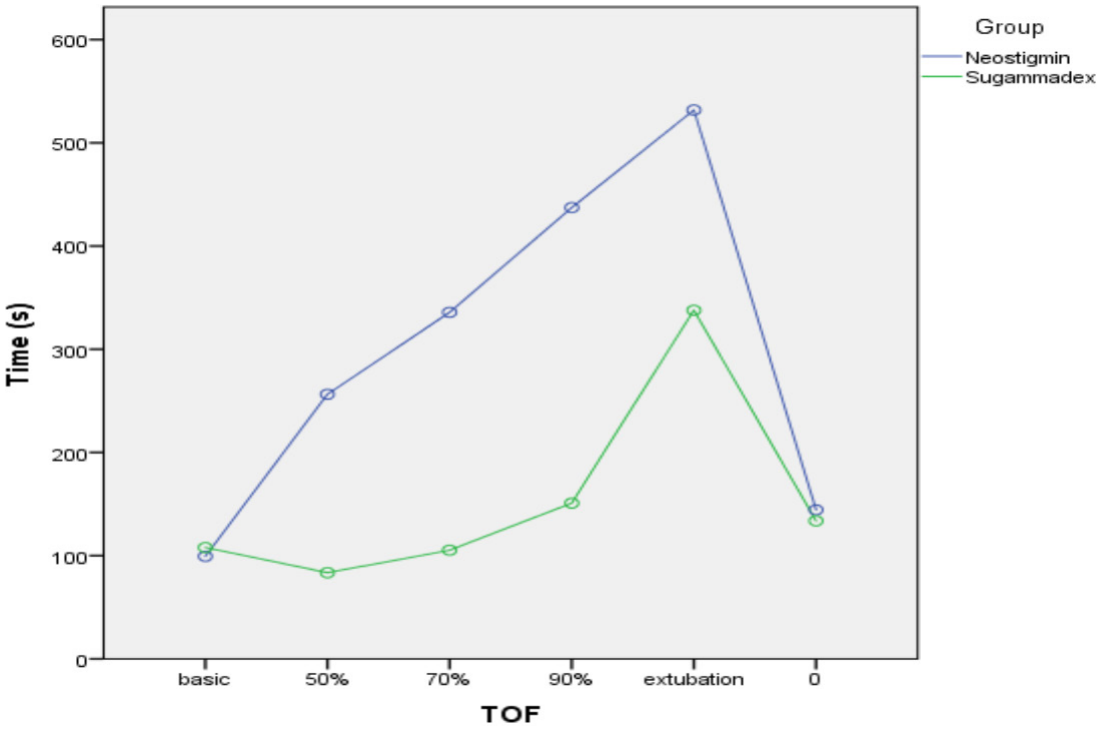
The graphics of the neostigmine (Group neostigmine and vitamin D < 30 ng/mL, n=31) and sugammadex (Group sugammadex and vitamin D < 30 ng/mL, n=37) when vitamin D status is under 30 ng/mL.

Sugammadex encapsulates the rocuronium and separates it from the receptor. When we examined our results, although the vitamin D levels below 30 ng/mL were different in number, the TOF 50% group (P = 0.015) and extubation (P = 0.007) were statistically significantly prolonged in the (Group sugammadex and vitamin D < 30 ng/mL, n=37) (Figures 1 and 2).

It is noteworthy that this difference is due to elongation in reaching the target at low vitamin D levels, but that there is no difference in neostigmine. We believe that more comprehensive studies with more patients are needed in this area. Studies showing that muscle weakness due to vitamin D deficiency, especially in proximal muscles and vitamin D supplementation have improved and constitute the main supporting base of our study [20–22]. Therefore, we can foresee that the muscle relaxation reversal agent can be affected by the mode of action and the reverse duration of vitamin D levels instead.

Our secondary outcome is the effect of low vitamin D levels on rocuronium time to TOF disappearance. We found that there was also no difference between the groups in terms of the time until the muscle relaxant was given and disappearance of TOF (TOF0) during the induction of anesthesia.

In conclusion, vitamin D deficiency is an important factor for anesthesiologists in terms of muscle strength and extubation time. In our study vitamin D deficiency seems to have prolonged the sugammadex extubation times and 50% TOF reach times but not effect on neostigmine reverse times. We think that more comprehensive studies with vitamin D replacement will give clearer results in this area in the future.

## Acknowledgment

The Düzce University Scientific Research Project Coordinating Department supported this work (2014.04.02.221).

## Abbreviations

25(OH)D: 25-Hydroxyvitamin D

1,25(OH)2 D: 1,25 Dihydroxyvitamin D

PTH: Parathyroid hormone

iPTH: intact parathyroid hormone

Mg: Magnesium

Ca: Calcium

FEV1: 1st minute force expiratory volume

FVC: Force vital capacity

TOF: Train of four

NMBAs: Neuromuscular blocking agents
